# A Different Approach to Estimate Temperature-Dependent Thermal Properties of Metallic Materials

**DOI:** 10.3390/ma12162579

**Published:** 2019-08-13

**Authors:** Luís Felipe dos Santos Carollo, Ana Lúcia Fernandes de Lima e Silva, Sandro Metrevelle Marcondes de Lima e Silva

**Affiliations:** Heat Transfer Laboratory—LabTC, Institute of Mechanical Engineering—IEM, Federal University of Itajubá—UNIFEI, Campus Prof. José Rodrigues Seabra, Av. BPS, 1303, Itajubá 37500-903, MG, Brazil

**Keywords:** temperature-dependent thermal properties, simultaneous estimation, optimization, sensitivity coefficients, uncertainty analysis

## Abstract

Thermal conductivity, *λ*, and volumetric heat capacity, *ρc_p_*, variables that depend on temperature were simultaneously estimated in a diverse technique applied to AISI 1045 and AISI 304 samples. Two distinctive intensities of heat flux were imposed to provide a more accurate simultaneous estimation in the same experiment. A constant heat flux was imposed on the upper surface of the sample while the temperature was measured on the opposite insulated surface. The sensitivity coefficients were analyzed to provide the thermal property estimation. The Broydon-Fletcher-Goldfarb-Shanno (BFGS) optimization technique was applied to minimize an objective function. The squared difference objective function of the numerical and experimental temperatures was defined considering the error generated by the contact resistance. The temperature was numerically calculated by using the finite difference method. In addition, the reliability of the results was assured by an uncertainty analysis. Results showing a difference lower than 7% were obtained for *λ* and *ρc_p,_* and the uncertainty values were above 5%.

## 1. Introduction

At present, globalization provides newer, faster, more reliable, and more accurate techniques to estimate thermal properties of materials depending on temperature. The cost of obtaining the parameters is another important issue, since it determines the reliability to compete in the internal and external markets. This paper proposes a technique that may be applied, for example, to accurately select, from the point of view of thermal properties, which materials will be employed in the manufacturing of heat exchangers. The methodology that leads to correct values of the thermal properties allows for saving of energy and other consequent environmental benefits; matters which, recently, have been largely considered. The machining process can be cited as an example for the aforementioned saving. A large amount of heat produced during the cutting process is dissipated to the tool holder. Knowing the values of the thermal conductivity of the tool and the tool holder leads to the correct choice of their material. Therefore, researchers have developed several procedures in this field [1,2].

A number of methods are available to estimate the thermal properties considering precision, speed, and cost, among other characteristics. In this context, Jannot et al. [3] presented a study in which the thermal conductivity of insulated materials was determined based on a pulsed method with a good precision, Xamán et al. [4] applied a guarded hot plate apparatus for the same purpose, and Thomas et al. [5] determined thermal conductivity and specific heat of insulated materials by applying a new experimental design. These thermal properties may be determined individually or together, and a great part of the estimations happen safely, precisely, and rapidly; however, few were used for temperature-dependent estimation or metallic materials. Other researchers present techniques that allow the estimation of only one temperature-dependent thermal property, for example, Aksöz et al. [6] estimated the thermal conductivity of Al-Cu alloys by using a radial heat flow apparatus and changed the initial temperature and Karimi et al. [7] determined the thermal conductivity of silver alloys by varying the temperature based on a linear heat flux apparatus. Recently, many researchers have presented techniques to simultaneously estimate temperature-dependent thermal properties, such as Sadeghi et al. [8] who determined thermal conductivity and diffusivity of SiC samples by applying the microwave heating process, Zamel et al. [9] who presented an improved firework algorithm to solve inverse problems allowing simultaneous estimation of properties of molten salt, and Öztürk et al. [10] who presented a method to estimate thermal conductivity and specific heat temperature-dependent of thermal protective fabric with good results. Other studies were performed [11,12,13,14,15,16], but none of them included the possibility of estimating thermal properties of metals depending on temperature simultaneously. Moreover, the experimental apparatus for most of these techniques is usually expensive. 

Thus, this work presents a technique to simultaneously determine volumetric heat capacity, *ρc_p_*, and thermal conductivity, *λ*, of AISI 1045 and AISI 304 samples; variables that depend on temperature. Some advantages of this method are the low cost, the precision, and the speed when compared with the techniques cited. Additionally, the uncertainty analysis presented in this work considers the influence of the numerical and experimental temperature errors and contact resistance. This work presents the betterments carried out concerning Carollo et al. [17].

## 2. Materials and Methods

### 2.1. Thermal Design Model

**Figure 1:**
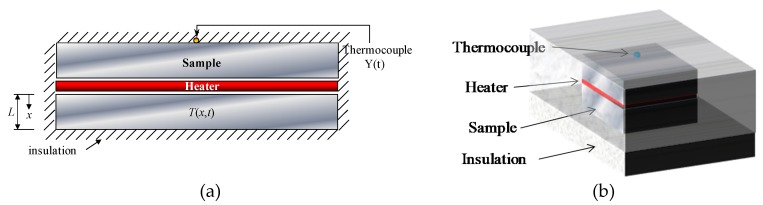
A one-dimensional (1D) representation of the model. (**a**) thermal model; (**b**) three-dimensional view of the thermal model.

The representation of the one-dimensional (1D) heat diffusion model is presented in Figure 1. This thermal model is obtained by using a resistive heater between two samples, and the sample-heater set is insulated. The thickness of the sample is much smaller than the other dimensions to ensure the one dimension.

The conduction equation for the problem in Figure 1 is:

(1) $$\frac{\partial }{\partial x}\lambda \left(T\right)\frac{\partial T\left(x,t\right)}{\partial x}=\rho c_{p}\left(T\right)\frac{\partial T\left(x,t\right)}{\partial t},$$

In accordance with the literature, there are two methods to estimate thermal properties dependent on temperature. In the first method, presented in Özisik [18], the thermal model is based on nonlinear heat conduction. The second method adopts constant thermal properties within a temperature range to solve the thermal model [19]. Thus, the initial temperature was defined (T_0_) and the estimation of the properties occurred considering 5 °C as the maximum range of temperature. This condition was performed for all the desired temperatures. 

Therefore, the heat diffusion equation for the thermal problem with constant thermal properties is expressed as below:

(2) $$\frac{\partial T\left(x,t\right)}{\partial x^{2}}=\frac{\rho c_{p}}{\lambda }\frac{\partial T\left(x,t\right)}{\partial t},$$

subjected to the following conditions of boundary:

(3) $$-\lambda \frac{\partial T\left(x,t\right)}{\partial x}=\varphi \left(t\right)\;at\;x=0,$$

(4) $$\frac{\partial T\left(x,t\right)}{\partial x}=0\;at\;x=L,$$

and the initial condition:

(5) $$T\left(x,t\right)=\;T_{0}\;at\;t=0,$$

where *x* is the heat direction, *t* the temporal interval, φ the applied heat flow, *T*_0_ the temperature in the beginning of the process, and *L* the thickness.

The finite difference method was used to calculate the numerical temperature of the conduction (Equation (2)).

### 2.2. Objective Function

Equation (6) presents the objective function applied to estimate *ρc_p_* and *λ*:

(6) $$F={\left(R_{c}^{\prime \prime }\varphi_{m}\right)}^{2}+\sum_{j=1}^{m}{\left(Y_{j}-T_{j}\right)}^{2},$$

where *m* is the number of points where temperature was measured, *Y* is the measured temperature, $R_{c}^{\prime \prime }$ is the heater thermal contact resistance, and $\varphi_{m}$ is the weighted average heat flow.

The optimum values of *ρc_p_* and *λ* are required to minimize Equation (6). To perform this procedure, the BFGS sequential optimization technique [20] is used in this work.

### 2.3. Experimental Procedure

**Figure 2:**
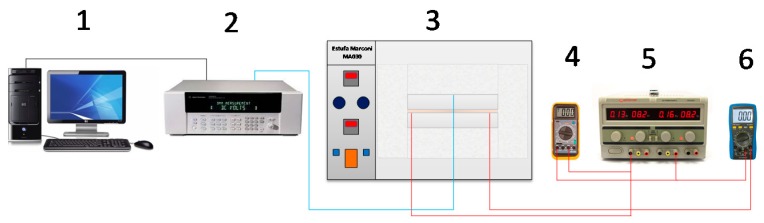
Experimental apparatus sketch used to estimate *ρc_p_* and *λ*.

The experimental apparatus sketch used is shown in Figure 2. As can be seen, all the components are numbered following this configuration: 1: Micro-computer; 2: Data acquisition; 3: Oven; 4: Multimeter; 5: Power supply; and 6: Multimeter. 

In this work, two materials were analyzed: AISI 1045 Steel (99.9 × 99.9 × 11.9 mm^3^) and AISI 304 stainless steel (49.9 × 49.9 × 10.5 mm^3^). Due to the different dimensions of the samples, a 99.5 × 99.5 × 0.2 mm^3^ resistive kapton heater with 23.2 Ω was necessary and another of 48.5 × 48.5 × 0.2 mm^3^ with 24.4 Ω. The resistive kapton heater was chosen due to its thinness, which allows a faster and more uniform warming. An Instrutemp ST-305 D-II power supply (Instrutemp Instrumentos de Medição Ltda, São Paulo, Brazil) was used to provide the adequate heat flow to perform the experiments. To ensure the correct values of current and resistance, calibrated multimeters were used (Minipa ET-2042-C (Minipa do Brasil, Joinvile, Brazil) for the current and Instrutherm MD-380 (Instrutherm Instrumentos de Medição, São Paulo, Brazil) for the resistance. Additionally, a symmetrical assembly was set up to reduce the errors caused by the measurement of heat flux on the top surface. A data acquisition system Agilent 34980A (Agilent, Santa Clara, CA, USA) was used to connect Type T thermocouples (30 AWG), welded by a capacitive discharge. To provide different initial conditions, the heater-sample set was placed inside a MA030 Marconi oven (Marconi Equipamentos para Laboratórios, Piracicaba, Brazil). The whole set was insulated by ceramic fiber plates with two purposes (Figure 1b): To guarantee a 1D heat flow and to reduce the convection effects. Lastly, all the experiments were performed in a temperature-controlled room.

## 3. Results and Discussion

All the experiments were performed following the procedure defined by Carollo et al. [17].

### 3.1. AISI 1045 Steel

In order to achieve significant results to simultaneously estimate *λ* and *ρc_p_*, 15 experiments were performed for each initial condition (25 °C, 50 °C, 75 °C, 100 °C, 125 °C, and 150 °C). The experiment lasted 80 s each, following these conditions: 0–10 s with heat flux of 7709 Wm^−2^; 10–70 s with 1854 Wm^−2^, and 70–80 s without heat flux. These conditions provide the best estimation of the thermal properties [17] and respect the hypotheses of constant thermal properties for each initial condition, since the difference between the initial and final temperature must be lower than 5 K. Lastly, the temperature was monitored in a time interval of 0.1 s so as to have more data.

To ensure the best region and the ideal condition to determine the thermal properties, two analyses were done: The first analysis corresponded to the sensitivity and objective function and the second concerned the best condition and design for the experiments [17].

**Figure 3:**
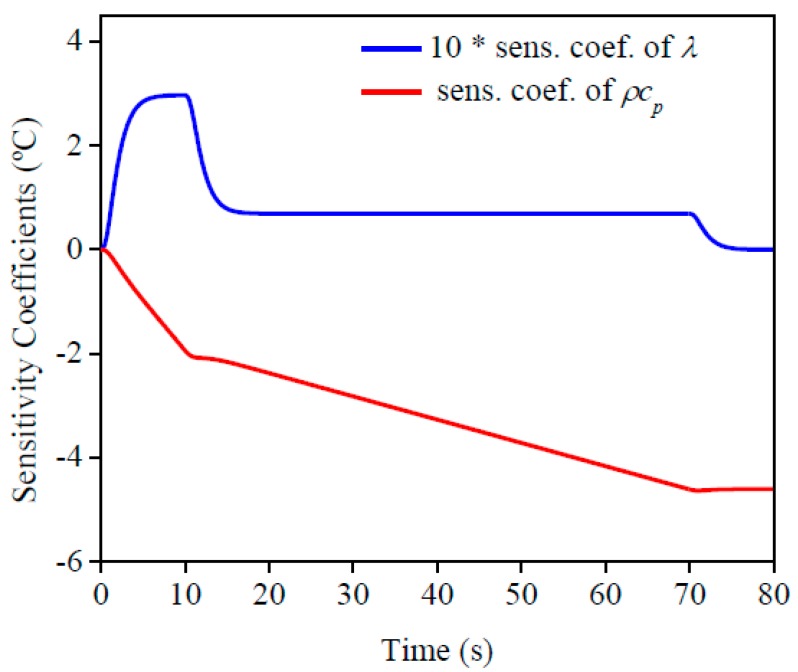
AISI 1045 steel with its corresponding sensitivity coefficients.

Figure 3 presents the sensitivity coefficient (sens. coef.) of *ρc_p_* and *λ* at *x* = *L*. These coefficients are important to indicate the best conditions to estimate the properties, such as, experimental time, time interval, number of points analyzed, and others. The sensitivity coefficient of *λ* increased only in the beginning of the experiment and remained constant after the heat flux was changed, until the power supply was turned off. By analyzing the sensitivity coefficient of *ρc_p_* it was possible to confirm that the value increased while there was heat flux. Because of this behavior, the best condition to simultaneously estimate the properties was applying two diverse heat flux intensities, the higher of which was applied in the beginning to maximize the sensitivity for thermal conductivity estimation and the lower was applied to guarantee enough sensitivity for the volumetric heat capacity estimation. It is important to claim that simultaneous estimation was possible because there was no dependence between the sensitivity curves.

**Figure 4:**
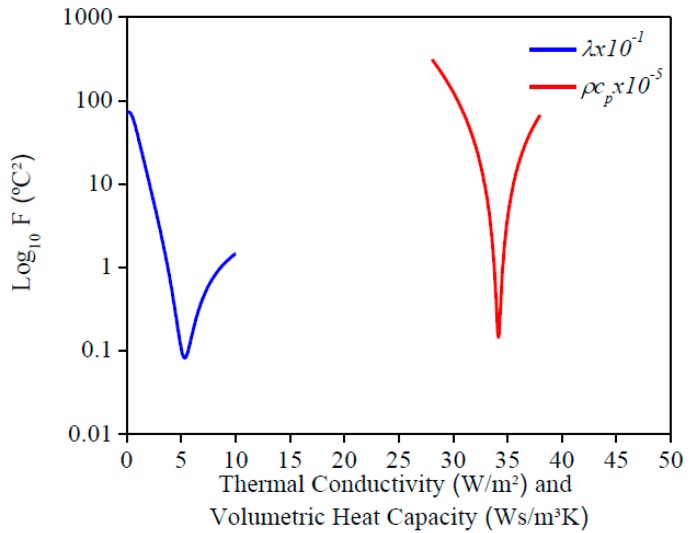
Values of *F* (Equation (6)) for AISI 1045 steel.

The evaluation of Equation (6) for each property is shown in Figure 4. *λ* and *ρc_p_* were estimated simultaneously due to a minimum value for each property. It is important to inform that the contact resistance of the heater on the sample was considered in Equation (6) to find its influence on the temperature measurements. In this study, a temperature difference of 0.23 °C corresponded to the influence of this contact resistance.

**Figure 5:**
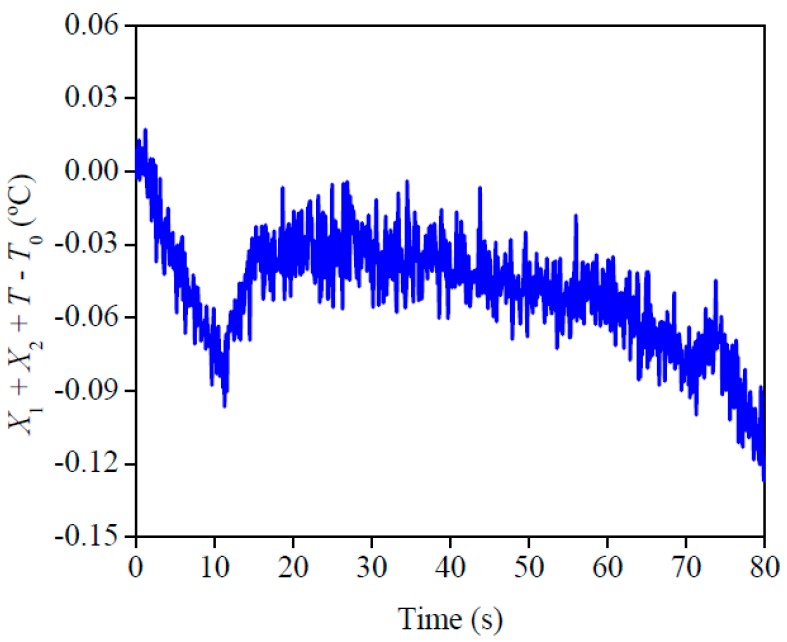
Results of the ideal condition and design for the experiment on AISI 1045.

The best condition and design for the experiment is presented in Figure 5. This analysis indicates that the best quality for the experiment was when the sensitivity coefficient of *ρc_p_* and *λ* plus the temperature difference was close to 0 (*X*_1_ + *X*_2_ + *Y* − *Y*_0_ ≅ 0). This analysis is relevant because it is a complement to the sensitivity analysis, in other words, it is a confirmation that all the established conditions allow a precise property estimation. A good condition of the experiment may be seen here, since the highest difference was around 0.12 °C. This affirmation can be checked when the obtained difference is compared with the difference between the final and initial temperature of each experiment, which is around 4 °C. 

**Figure 6:**
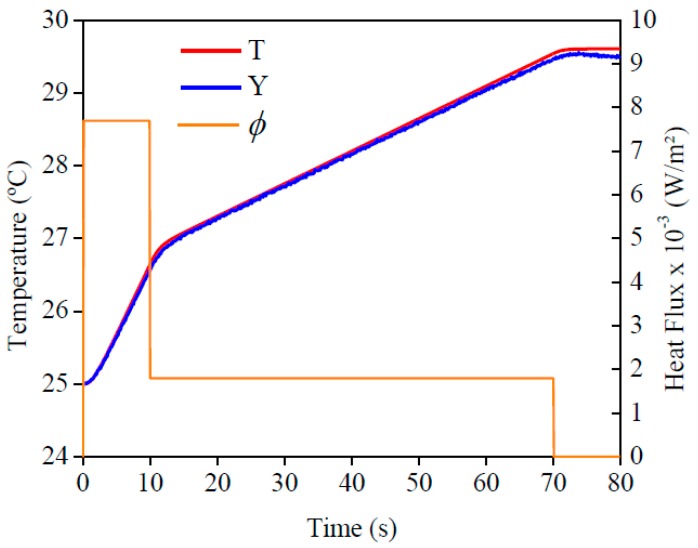
Experimental heat flow (ϕ) for AISI 1045. Comparison of temperatures obtained numerically (*T*) and experimentally (*Y*).

**Figure 7:**
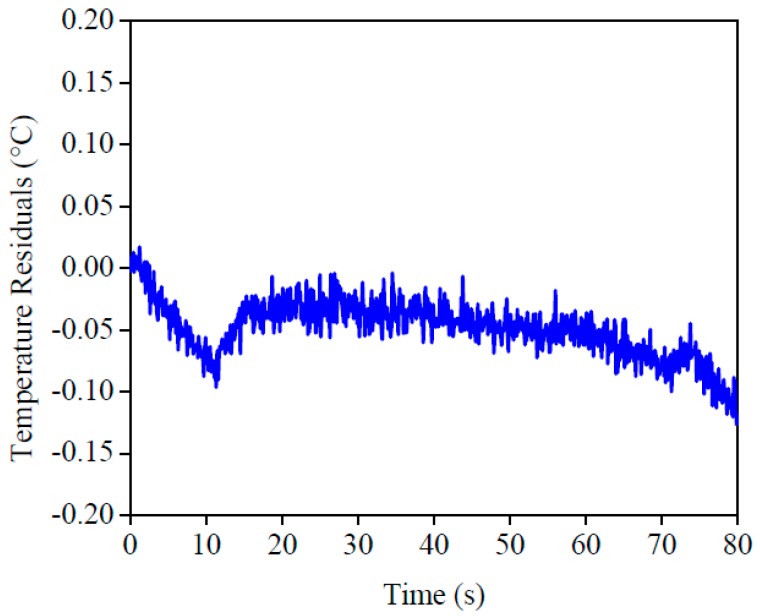
Residuals of temperatures of AISI 1045.

Figure 6 shows the applied heat flux at *x* = 0 and the temperatures values at *x* = *L*. It is possible to see the good agreement between the experimental temperature and numerical temperature, which was calculated by using the obtained thermal properties. To confirm this affirmation, Figure 7 presents the residuals between these temperatures. Once the maximum difference found was around 0.10 °C, it was possible to confirm the good quality of the methodology. This affirmation can be validated by comparing the obtained difference with the thermocouple uncertainty, that it is around 0.10 °C. Lastly, this small difference can be attributed to the isolation condition.

The results of *ρc_p_* and *λ* on the AISI 1045 steel sample for each initial temperature are presented in Table 1. The percentage difference between the average and the literature value was considered to calculate the error. Based on the lower standard deviation and the error found, the estimated values of *ρc_p_* and *λ* show a good conformity when compared to the literature values. One can also see that the results of *ρc_p_* are more precise due to its higher sensitivity (Figure 3).

**Figure 8:**
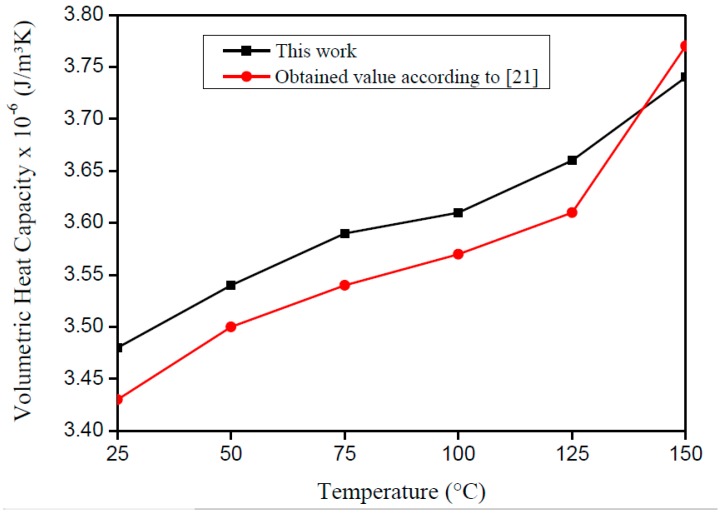
Comparison between the literature values with the estimated results of *ρc_p_* on the AISI 1045 steel plate.

**Figure 9:**
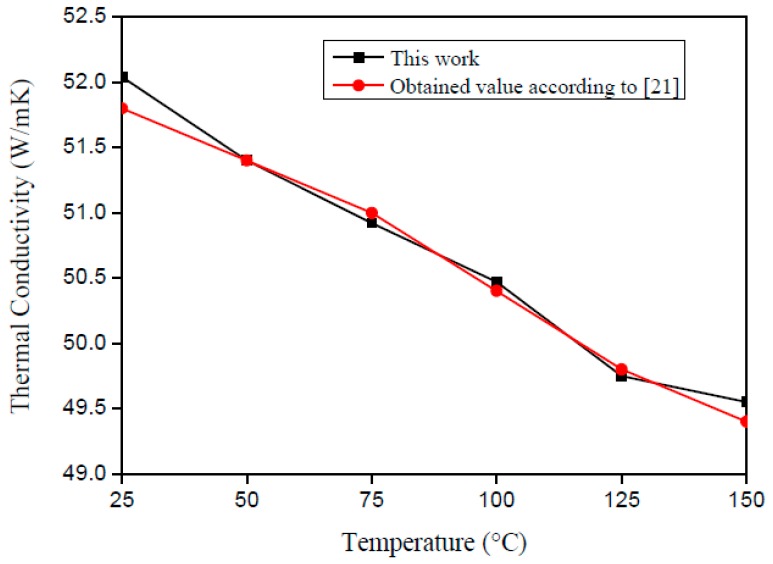
Comparison between the literature values with the estimated results of *λ* on the AISI 1045 steel sample.

Figure 8 and Figure 9 present the literature and experimental result values. One can see the good agreement of the curves, which present a correlation factor of 0.98 for *λ* and 0.99 for *ρc_p_*. In accordance with Montgomery and Runger [22], the correlation factor indicates a quantitative measurement between two factors. Moreover, when the correlation factor presents value from +0.9 up to +1.0, it is possible to say that the correlation is direct and reliable.

From these results, Equations (7) and (8) can be written as follows for *ρc_p_* and *λ*, respectively:

(7) λ(T)=−0.02228×T+52.500 [W/mK],

(8) $$\rho c_{p}\left(T\right)=\left(0.00210\times T+3.43253\right)\times 10^{6}\;\left[J/m^{3}K\right],$$

These equations can be used in the range of 25 °C up to 150 °C.

### 3.2. AISI 304 Stainless Steel

Following the same procedure that was applied for the AISI 1045 steel sample, 15 experiments were performed for each initial condition (25 °C, 50 °C, 75 °C, 100 °C, 125 °C, and 150 °C) to estimate *ρc_p_* and *λ* simultaneously. Each experiment lasted 150 s following this condition: 0–20 s with a heat flux of 2672 Wm^−2^; 20–140 s with 668 Wm^−2^, and 140–150 s without a heat flux. 

**Figure 10:**
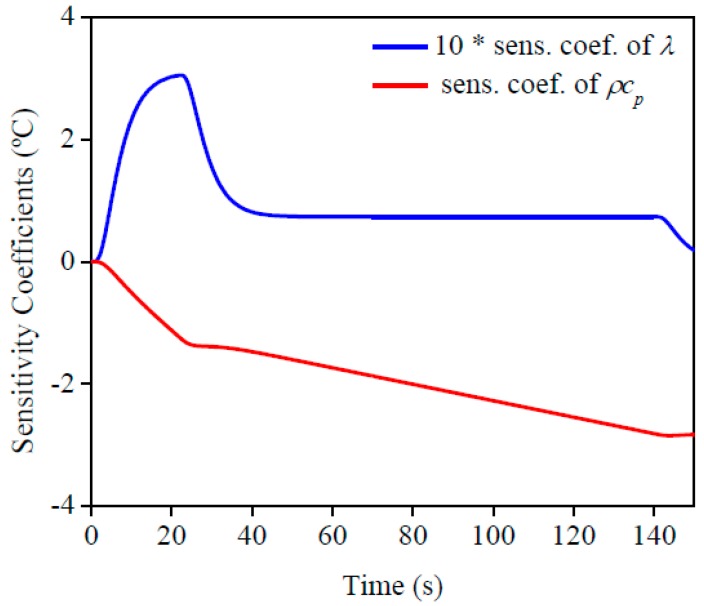
Sensitivity coefficient of the AISI 304 stainless steel sample.

**Figure 11:**
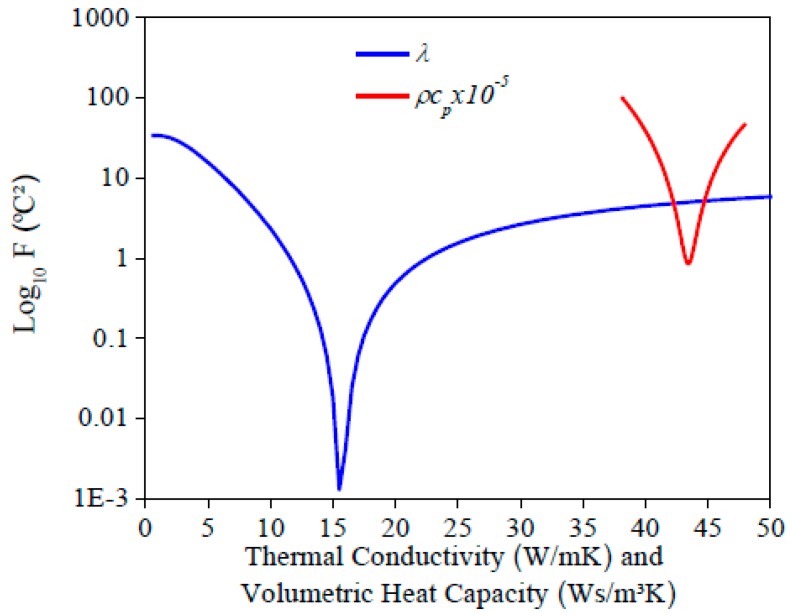
Values of the objective function for the AISI 304 sample.

Figure 10 and Figure 11 present the sensitivity coefficients and the objective function, respectively, for each property. It can be seen that the behavior found was the same as the AISI 1045 steel, so it is possible to estimate the properties simultaneously. By analyzing the sensitivity coefficient of both materials, it is possible to affirm that the thermal conductivity estimation could be more precisely for AISI 304 stainless steel than 1045 steel. This is because there is more time for information, in other words, more points to analyze, and the difference between the values of the sensitivity coefficients for both properties are lower. This behavior is a consequence of the lower thermal conductivity of stainless steel when it is compared to 1045 steel. 

**Figure 12:**
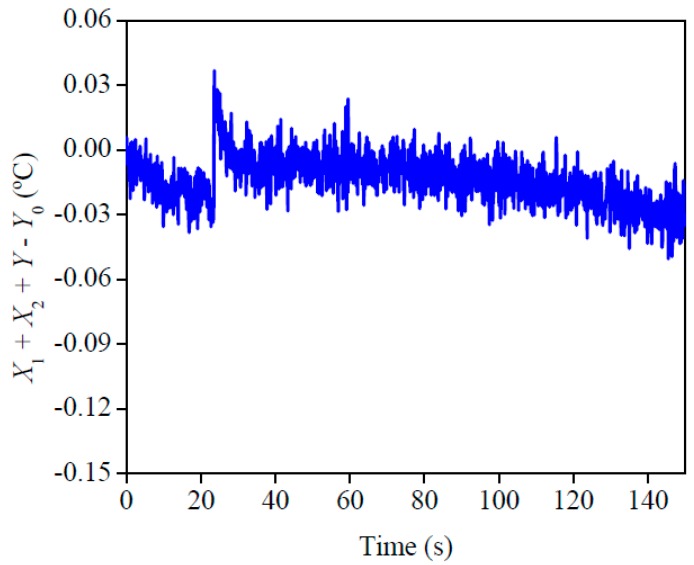
Results of the ideal condition and design for the experiment with AISI 304.

Figure 12 shows the results for the analysis of the best experimental configuration. The maximum deviation found, around 0.05 °C, was lower than the uncertainty of the thermocouple, which confirms the reliability of the results and the good experimental configuration defined based on Figure 10 and Figure 11.

**Figure 13:**
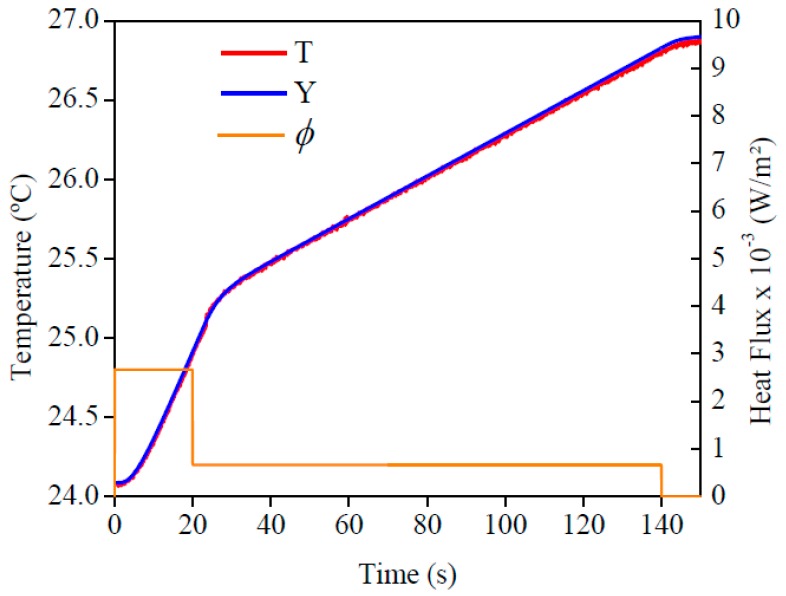
Experimental heat flow (ϕ) for AISI 304. Comparison of temperatures obtained numerically (*T*) and experimentally (*Y*).

**Figure 14:**
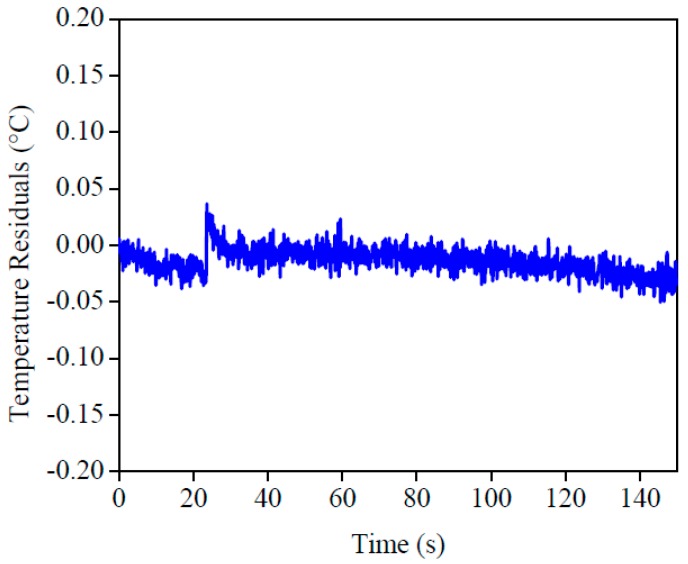
Residuals of temperature of AISI 304.

The imposed heat flux and the temperatures are presented in Figure 13. By analyzing this figure, one can see the good concordance between the temperatures. To validate this affirmation, the temperature residuals, where the maximum deviation was 0.05 °C, are presented in Figure 14. If this value was compared to the temperature difference, around 3 °C, one could see the good quality of the obtained results. 

The results of *ρc_p_* and *λ* on the AISI 304 stainless steel sample for each initial temperature are presented in Table 2. Based on the lower standard deviation and the error found, the estimated values of *ρ*c_p_ and *λ* show a good conformity when compared to the literature values. This affirmation is based on the low standard deviation and error found. Similar to AISI 1045 steel, one may see that the results of *ρc_p_* are more precise due to its higher sensitivity.

**Figure 15:**
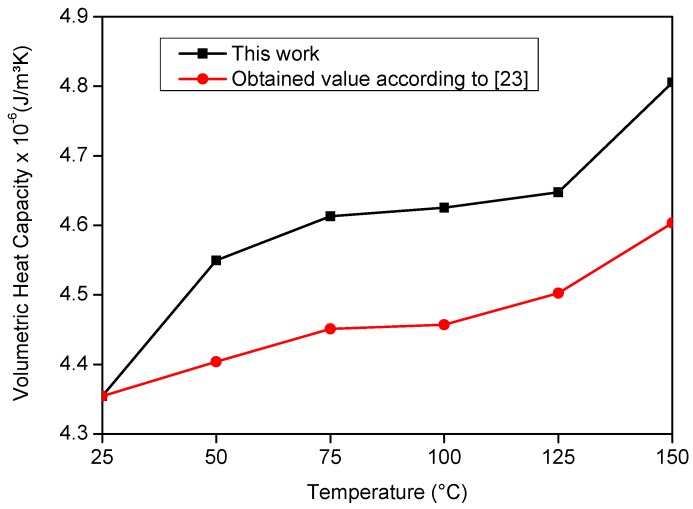
Comparison between the literature values with the estimated results of *ρc_p_* on the AISI 304 stainless steel sample.

**Figure 16:**
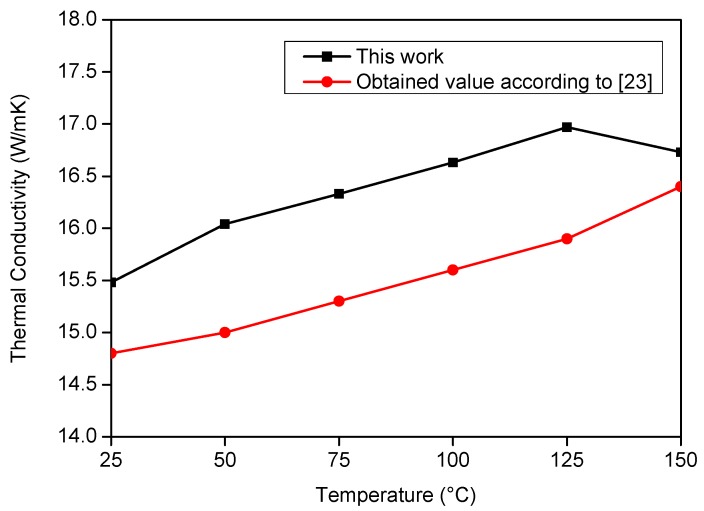
Comparison between the literature values with the estimated results of *λ* on the AISI 304 stainless steel sample.

Figure 15 and Figure 16 present the literature and experimental result values. On analyzing Figure 15 and Figure 16, it is possible to verify the good agreement between the obtained results and those from the literature. To validate this affirmation, a correlation study was performed, and the correlation factor was 0.86 for *λ* and 0.95 for *ρc_p_*.

From these results, Equations (9) and (10) can be written as follows for *ρc_p_* and *λ* respectively:

(9) λ(T)=0.01067×T+15.4293 [W/mK],

(10) $$\rho c_{p}\left(T\right)=\left(0.0029266\times T+4.34313\right)\times 10^{6}\;\left[J/m^{3}K\right],$$

These equations can be used in the range from 25 °C to 150 °C.

## 4. Uncertainty Analysis

The uncertainty propagation was considered to perform this analysis, as described in Carollo et al. [17] and Taylor [24], and it is important to assure the reliability of the estimated results.

Equations (11) and (12) show the uncertainty estimation based on the objective function (Equation (6)):

(11) $$U_{final}^{2}=U_{Y}^{2}+U_{T}^{2}+U_{BFGS}^{2},$$

(12) $$U_{final}^{2}=U_{aquis.}^{2}+U_{therm.}^{2}+U_{contact\;resist.}^{2}+U_{insul.}^{2}+U_{current}^{2}+U_{resistance}^{2}+U_{MDF}^{2}+U_{BFGS}^{2}$$

Individual uncertainty, which was divided by the mean value of the parameter, was used to calculate the partial uncertainty. Therefore, Table 3 presents the final uncertainty. One can see that these values are acceptable once they are around 5%.

## 5. Conclusions

This paper presents a different approach for the estimation of *λ* and *ρc_p_* in metallic materials simultaneously depending on temperature. The materials analyzed were the AISI 304 stainless steel and AISI 1045 steel. The good results found can be confirmed since the difference between the estimated values and literature is small, that is, lower than 7%, the standard deviation is the low, and the good uncertainty values are lower than 6%. 

This work is validated to estimate *λ* and *ρc_p_* simultaneously in metals. However, this technique may be applied to reliably estimate *λ* and *ρc_p_* of metals that present thermal conductivity from 10 W/mK to 60 W/mK in a range of 25 °C up to 150 °C.

For future work, the use of a thermal model designed in three dimensions should be used to analyze the locations of temperature sensors in different positions to determine the areas that display better sensitivity to estimate *λ* and *ρc_p_*.

## Figures and Tables

**Table 1:** Average results, standard deviation, and error of *ρc_p_* and *λ* for AISI 1045.

Average T_0_ (°C)	Thermal Properties	Mean	Obtained value from Grzesik et al. [21]	Standard Deviation	Error (%)
25.4	*ρc_p_* × 10^−6^ (J/m³K)	3.48	3.43	±0.02	1.44
	*λ* (W/mK)	52.04	51.80	±0.49	0.46
50.1	*ρc_p_* ×10^−6^ (J/m³K)	3.54	3.50	±0.05	1.18
	*λ* (W/mK)	51.39	51.4	±0.25	0.02
75.4	*ρc_p_* × 10^−6^ (J/m³K)	3.59	3.54	±0.04	1.36
	*λ* (W/mK)	50.92	51.00	±0.37	0.16
100.4	*ρc_p_* × 10^−6^ (J/m³K)	3.61	3.66	±0.03	0.99
	*λ* (W/mK)	50.47	50.40	±0.28	0.14
125.3	*ρc_p_* × 10^−6^ (J/m³K)	3.66	3.61	±0.05	1.42
	*λ* (W/mK)	49.75	49.80	±0.38	0.10
150.2	*ρc_p_* × 10^−6^ (J/m³K)	3.74	3.77	±0.02	0.86
	*λ* (W/mK)	49.55	49.40	±0.36	0.30

**Table 2:** Results obtained for the AISI 304 stainless steel sample.

Average T_0_ (°C)	Thermal Properties	Mean	Obtained value from Abas et al. [23]	Standard Deviation	Error (%)
24.8	*ρc_p_* × 10^−6^ (J/m³K)	4.35	4.35	±0.12	0.03
	*λ* (W/mK)	15.49	14.8	±0.39	4.64
49.7	*ρc_p_* × 10^−6^ (J/m³K)	4.55	4.40	±0.09	3.31
	*λ* (W/mK)	16.04	15.0	±0.58	6.95
74.9	*ρc_p_* × 10^−6^ (J/m³K)	4.61	4.45	±0.06	3.64
	*λ* (W/mK)	16.33	15.3	±0.47	6.76
99.2	*ρc_p_* × 10^−6^ (J/m³K)	4.63	4.46	±0.07	3.77
	*λ* (W/mK)	16.63	15.6	±0.53	6.62
124.9	*ρc_p_* × 10^−6^ (J/m³K)	4.65	4.50	±0.06	3.22
	*λ* (W/mK)	16.97	15.9	±0.57	6.74
149.5	*ρc_p_* × 10^−6^ (J/m³K)	4.80	4.60	±0.09	4.38
	*λ* (W/mK)	16.73	16.4	±0.67	2.01

**Table 3:** Uncertainty values for each analyzed material.

Material	Uncertainty (%)
AISI 1045 steel	5.45
AISI 304 stainless steel	4.79
